# Biocontrol of Soft Rot of Chinese Cabbage Using an Endophytic Bacterial Strain

**DOI:** 10.3389/fmicb.2019.01471

**Published:** 2019-07-03

**Authors:** Wenyan Cui, Pengjie He, Shahzad Munir, Pengbo He, Yueqiu He, Xingyu Li, Lijuan Yang, Biao Wang, Yixin Wu, Pengfei He

**Affiliations:** ^1^Guizhou University of Traditional Chinese Medicine, Guiyang, China; ^2^Faculty of Plant Protection, Yunnan Agricultural University, Kunming, China; ^3^National and Local Joint Engineering Research Center for Screening and Application of Microbial Strains, Kunming, China; ^4^Faculty of Agronomy and Biotechnology, Yunnan Agricultural University, Kunming, China; ^5^Faculty of Science, Yunnan Agricultural University, Kunming, China

**Keywords:** *Pectobacterium carotovorum* subsp. *carotovorum*, *Bacillus amyloliquefaciens*, bio-organic substrate, rhizosphere competence, colonization, biocontrol

## Abstract

Soft rot caused by *Pectobacterium carotovorum* subsp. *carotovorum* (*Pcc*) is a major constraint in the production of Chinese cabbage. The objective of this study was to demonstrate that the causative agent *Pcc* may be successfully managed by *Bacillus amyloliquefaciens* KC-1, both *in vitro* and *in vivo*. Chinese cabbage seedlings were cultivated in organic substrate termed bio-organic substrate using a floating-seedling system with *B. amyloliquefaciens* KC-1. This approach was applied in a greenhouse to evaluate the management of soft rot. The results showed that the extent of soft rot, as well as the transmission of *Pcc* to the stem progeny and its survival in the rhizosphere, was reduced following inoculation with *B. amyloliquefaciens* KC-1. In contrast, the population diversity of *B. amyloliquefaciens* KC-1 persisted in the Chinese cabbage stems after germination. These findings revealed that *B. amyloliquefaciens* KC-1 was able to survive and suppress the growth of *Pcc* in Chinese cabbage and its rhizosphere, protecting the host from the pathogen. The use of *B. amyloliquefaciens* KC-1 throughout the growth period of plants may be an effective strategy for the prevention of soft rot in Chinese cabbage.

## Introduction

*Pectobacterium carotovorum* subsp. *carotovorum* (*Pcc*) is a common cause of soil-borne soft rot, in a broad range of vegetable and flower hosts such as Chinese cabbage, tomato, potato, cucumber, *Amorphophallus konjac*, and *Zantedeschia hybrida* (Des Essarts et al., 2016; Kang et al., 2016; Shao et al., 2016; Garge and Nerurkar, 2017; He et al., 2018). The typical symptoms include maceration and rotting of leaves and other organs of plant, resulting in loss of the marketable yield. Furthermore, soft rot may occur during transit, storage, or marketing (Bhat et al., 2010).

Soil-borne diseases are difficult to overcome. Chemical methods – though generally effective – are not desirable due to concerns regarding the development of resistance and environmental pollution (Gill and Garg, 2014). Effective and environmentally friendly methods for controlling these diseases are required to reduce the use of chemical pesticides. Biocontrol is one of the most effective and promising approaches for the control of soft rot and other plant diseases. Previous studies revealed a limited number of bacteria (i.e., *Bacillus*, *Actinomycete*, *Pseudomonas*, *Lactobacillus*, and *Serratia*), which may be useful as biocontrol agents against soft rot pathogens (El Karkouri et al., 2010; Czajkowski et al., 2012; Des Essarts et al., 2016; Tsuda et al., 2016; Garge and Nerurkar, 2017; Gerayeli et al., 2018). Among these, *Bacillus subtilis* and *Bacillus amyloliquefaciens* (*B. amyloliquefaciens*) have attracted considerable attention owing to its strong biological control activity and remarkable environmental suitability to survive under adverse conditions (Romero et al., 2007; Ongena and Jacques, 2008).

*Bacillus amyloliquefaciens* suppresses the pathogens through competition, promotion of growth, antibiosis, and induction of systemic resistance (Diallo et al., 2011). The polyketides (i.e., difficidin, bacillaene, and macrolactin) of non-ribosomal synthesis are the most effective antibacterial compound constituents by *B. amyloliquefaciens* and *B. subtilis* (Chen et al., 2009; Chowdhury et al., 2015). Another product of non-ribosomal synthesis, the dipeptide bacilysin consisting of anticapsin and alanine moieties, together with the polyketides, was found as being involved in contribute the control of bacterial plant diseases (Bais et al., 2004; Chen et al., 2009; Zeriouh et al., 2011). However, the effectiveness of endophytes as biological control agents (BCAs) is dependent on efficient colonization of the plant environment. The extent of endophytic colonization in plant rhizosphere and tissues reflects the ability of bacteria to selectively adapt and compete in those specific ecological niches (Chowdhury et al., 2015; Des Essarts et al., 2016; Abdallah et al., 2018; Munir et al., 2018).

The majority of biocontrol agents against *Pcc* are applied to the rhizosphere using water. Rhizosphere competence is a prerequisite for the effective biological control of soft rot. Furthermore, stable biological control against soil-borne pathogens cannot be expected merely through the application of an antagonist without a suitable management method. This is because the introduced biocontrol agents compete for niches and nutrients with other native microbes for their survival (Kokalis–Burelle et al., 2002; El-Hassan and Gowen, 2006).

In this study, we isolated *B. amyloliquefaciens* KC-1 from an infected field of Chinese cabbage (cv. “Qingdao 83-1”) to control soft rot. We used biological and molecular tools to identify and characterize the bacterium. In addition, we assessed its effectiveness in terms of biological control against the transmission of soft rot pathogen *Pcc* through experiments in the greenhouse. The aim of this research was to establish effective management strategies for the development of a safe microbial pesticide applicable to the production of Chinese cabbage in the greenhouse and field.

## Materials and Methods

### Bacterial Strains, Growth Conditions, and Preparation of Inoculum

All bacterial strains were grown in Luria-Bertani (LB) medium (NaCl, 10 g/l; bacto tryptone, 5 g/l; yeast extract, 5 g/l; AoBoXing Bio-tech Co., Ltd., Beijing, China) at 30 (*B. amyloliquefaciens*) and 28°C (*Pcc*) for 48 h under shaking (160 rpm). Whenever necessary, bacteria were washed thrice using isotonic saline solution (NaCl, 8 g/l) through centrifugation at 5,000 rpm for 5 min, followed by resuspension of the pellets in saline. *Pcc* strain E1 had been isolated from symptomatic Chinese cabbage and identified (MH934929).

### Isolation and Identification of *B. amyloliquefaciens* KC-1

*Bacillus amyloliquefaciens* KC-1 was isolated from the rhizosphere of an asymptomatic Chinese cabbage plant infected with soft rot in Kunming City, China (25°13′ N, 102°75′ E). The physiological and biochemical properties as well as the morphological characteristics were analyzed as previously described (Logan and De Vos, 2009). The amplification and sequencing of the *gyr*B gene was conducted using the following universal primers: GyrB-F (5′-GAA GTC ATC ATG ACC GTT CTG CAY GCN GGN AAR TTY GA-3′) and GyrB-R (5′-AGC AGG GTA CGG ATG TGC GAG CCR TCN ACR TCN GCR TCN GTC AT-3′) (Abdallah et al., 2018). The obtained sequences were compared with known sequences of the bacterial *gyr*B gene available in the National Center for Biotechnology Information database using the BlastT program^1^. Furthermore, a phylogenetic tree was constructed using the neighbor-joining method with the MEGA (version 7.0) software (MEGA, United States) (Kumar et al., 2016).

### *In vitro* Antibacterial Activity of *B. amyloliquefaciens* KC-1 Against *Pcc*

Testing of *in vitro* antibacterial activity was performed using LB agar plates (Des Essarts et al., 2016) in quadruplicates. *Pcc* E1 cell suspension [10^6^ colony-forming units (CFU)/ml] (200 μl) was evenly spread onto the surface of the LB plates (ϕ9.0 cm). Subsequently, 10 μl of *B. amyloliquefaciens* KC-1 culture and sterile distilled water (SDW) were added onto the edge of the plate. The plates were incubated at 30°C for 48 h.

### Biocontrol Activity of *B. amyloliquefaciens* KC-1 Against *Pcc* in Chinese Cabbage

The biological assays were performed as previously described (Dong et al., 2004). *B. amyloliquefaciens* KC-1 and *Pcc* E1 (10^8^ CFU/ml, each) were mixed as an inoculant. Healthy Chinese cabbages (1-month-old seedlings of Chinese cabbage, cv. “Qingdao 83-1”) were purchased. After sterilization of the surface, cabbage petioles were cut into pieces (∼3.5–4.0 × 5.5–6.5 cm in size). Subsequently, inoculant (10 μl) was inoculated onto the surface of the cabbage petioles (i.e., quadruplicate per condition) through a pipette without wounding. SDW, *B. amyloliquefaciens* KC-1, and *Pcc* E1 cell suspension were used separately here, respectively, as a control. The infected discs were placed in 17-cm sterile Petri dishes and incubated at 30°C for 24 h. The extent of soft rot on each disc was evaluated by detecting the maceration area and the percentage maceration (PM) (Dong et al., 2004). The maceration area in all cases was analyzed using Mshot Digital Imaging System (version 9.3.3.1) software (Mingmei, Guangzhou, China), PM = the weight of macerated tissue after inoculation × 100/the weight of tissue before inoculation. The data for the values of maceration area and PM were compared according to Duncan’s multiple range test, and a pairwise comparison test was performed. A *p* < 0.05 denoted statistical significance in all tests (SPSS software, version 22.0, Chicago, IL, United States).

In addition, 10 μl of inoculant solution, pure *B. amyloliquefaciens* KC-1, and inactivated *Pcc* E1 suspension were inoculated onto the surface of petioles of healthy Chinese cabbage in the greenhouse, as described above. The symptoms of soft rot were observed and evaluated after inoculation and growth in the greenhouse at 22–28°C for 3 days.

### *In vitro* Co-culture Assay

Overnight culture of *B. amyloliquefaciens* KC-1 (10^8^ CFU/ml) was used to initiate co-culture with *Pcc* E1 (10^8^ CFU/ml) in LB medium and incubated at 28°C at 150 rpm for 12 h. Similarly, *Pcc* E1 was inoculated and cultured alone as control to monitor the growth rate. The CFU of the co-cultured strains were recorded separately based on their distinct colony morphologies on the LB plates. Each treatment was performed in triplicate. The independent *t*-test (*p* < 0.05) was used to compare the CFU values of *B. amyloliquefaciens* KC-1 and *Pcc* E1 between strains grown alone and co-cultured.

### PCR Amplification of Antibiotic Genes of Nonribosomal Peptide Synthetase and Polyketide Synthetase

The amplification and sequencing of the genes involving in synthesis of the polyketides difficidin, bacillaene, and macrolactin and the dipeptide bacilysin were performed using the primers listed in Table 1. The PCR sample mix was prepared as follows. Initially, 2 μl of EasyTaq buffer (10×) (TransGen, Beijing, China) was vortexed with 1.6 μl of dNTPs (2.5 mM), 1 μl of forward and reverse primers (10 μM), and 0.5 μl of EasyTaq DNA polymerase (TransGen, Beijing, China). Extracted DNA (50 ng) from the bacterium (HiPure Bacterial DNA Kit, Magen, China) sample and about 14.0 μl SDW were added to the PCR reaction mixture in a total volume of 20 μl. The PCR was performed in a WD-9402A Thermal Cycler (Applied Biosystems, Beijing, China) as follows: denaturation step (5 min at 94°C), followed by 30 cycles of 40 s at 94°C, 40 s at 57°C, and 45 s at 72°C, and a final extension of 10 min at 72°C. PCR products were sequenced and analyzed as above.

**TABLE 1:** The PCR detection of the polyketides and the dipeptide biosynthesis genes from *B. amyloliquefaciens* KC-1.

	**Primers**	**Sequences (5′–3′)**	**PCR production**	**Gene**	**References**
Difficidin	dfnA-F	GGA TTC AGG AGG GCA TAC CG	653	*dfnA*	Compaoré et al.,2013
	dfnA-R	ATT GAT TAA ACG CGC CGA GC			
Bacillaene	baeA-F	ATG TCA GCT CAG TTT CCG CA	688	*baeA*	Compaoré et al.,2013
	baeA-R	GAT CGC CGT CTT CAA TTG CC			
Macrolactin	mlnA-F	CCG TGA TCG GAC TGG ATG AG	668	*mlnA*	Compaoré et al.,2013
	mlnA-R	CAT CGC ACC TGC CAA ATA CG			
Bacilysin	bacA-F	CAG CTC ATG GGA ATG CTT TT	498	*bacA*	Mora et al.,2011
	bacA-R	CTC GGT CCT GAA GGG ACA AG			

### Construction of Green Fluorescent Protein (GFP)-Tagged *B. amyloliquefaciens* KC-1

The GFP-tagged *B. amyloliquefaciens* KC-1 (KC-1-*gfp*) strain was obtained through conjugal transfer of the pHT01-P43GFPmut3a plasmid – carrying a GFP gene – into the KC-1 cytoplasm (He, 2014; Zhang et al., 2015). To assess the stability of the KC-1-*gfp* strain without selection, the KC-1-*gfp* was cultured overnight in LB medium (10 μg/ml chloramphenicol), followed by adjustment to a suspension (optical density [OD]_600_ = 1.0) in LB broth. Subsequently, the stability was evaluated through continuous culturing in fresh LB broth (0.1% w/w, per 5 h) without chloramphenicol for 60 h at 37°C under shaking (160 rpm). Furthermore, the suspension of KC-1-*gfp* and wild-type KC-1 were prepared as described above. Subsequently, 1.0 ml of suspension was introduced to 100 ml of fresh LB broth and cultured under the aforementioned conditions. During the initial 12 h post-inoculation, the OD_600_ of the culture was measured every 2 h at OD_600_. Between 12 and 28 h, the OD_600_ was measured every 4 h. Moreover, between 28 and 60 h, the OD_600_ was measured every 8 h.

### Preparation of Bio-Organic Substrate

For the following experiments in the greenhouse, *B. amyloliquefaciens* KC-1-*gfp* strain was used instead of the wild-type strain. KC-1-*gfp* was incubated and resuspended in SDW as described above. The seedling substrate was initially moistened using the KC-1-*gfp* suspension. The bacteria concentration was 10^7^ CFU/g. SDW at an equal volume was used as a negative control.

### Biocontrol of Chinese Cabbage Soft Rot in Greenhouse Assays

Four independent greenhouse assays were performed to assess the effectiveness of biocontrol against soft rot in cabbages over a period of 2 years (2015–2016). Two different greenhouses (25–30°C) located in Kunming city were used – one located at the NLJERCSAMS-YCFCL experiment site (Qinglong, Anning, Kunming, China) and the other at the FPP-YAU experiment site (Panlong, Kunming, China). The assays were termed according to their location and year as follows: A1 and A2 (2015 and 2016 in Anning, respectively) and P1 and P2 (2015 and 2016 in Panlong district, respectively).

A floating-seedling system was employed to cultivate the Chinese cabbage seedlings. The bio-organic substrate was gently spread on the foam trays (80 cm [L] × 50 cm [W] × 15 cm [H]). After sterilization of the surface (i.e., soaked in 70% ethanol for 2 min, immersed in 10% sodium hypochlorite for 5 min, and rinsed four times with SDW to remove residues), Chinese cabbage (Qingdao 83-1) seeds were sown in the commercial substrate, and trays were placed into a floating tank for 25 days in the greenhouse. The shoot height, fresh weight, and dry weight of 30 seedlings randomly selected were measured in triplicates prior to transplantation. The independent *t*-test (*p* < 0.05) was used to compare the values of shoot height, fresh weight, and dry weight between seedlings grown with bio-organic substrate and organic substrate.

A1 and A2 contained 80 plants per condition, while P1 and P2 contained 72 plants per condition. All experiments were set in a completely randomized design with six replicates under the following six conditions: seeds were directly sown in uninfected soil; seeds were directly sown in infected soil (10^7^ CFU/ml of E1 suspension, 100 ml/plot); seedlings were grown in organic substrate or bio-organic substrate using the floating-seedling system; and after transplantation, plants were infected with *Pcc* E1 alone and in combination with biocontrol strain KC-1-*gfp*. The biocontrol and pathogenic bacteria were inoculated separately. Two days after the transplantation of seedlings, the pathogen *Pcc* E1 was inoculated at 10^9^ CFU/plant onto transplanted plants through drenching with 100 ml cell suspension (10^7^ CFU/ml). Four days after the transplantation of seedlings, 100 ml KC-1-*gfp* suspension (10^7^ CFU/ml) was introduced to the plant petioles through watering. For each treatment – except for the uninfected plants – the plants were inoculated a total of four times at 7-day intervals after transplantation. Fertilizer, insecticides, and fungicides were applied according to the instructions provided by the manufacturers. At 70 days after transplantation, the incidence rate (IR), relative disease severity index (DSI), and protection value (PV) under each condition were used to determine the relative severity of the disease as follows (Tsuda et al., 2016): 0 = no symptoms; 1 = very small lesions on the outer leaves; 2 = rot on the outer leaves; 3 = rot on the outer leaves and part of the head; and 4 = rot on most parts of the head. IR = Σ(the number of diseased plants) × 100/total number of plants), DSI = Σ(scale × the number of diseased plants) × 100/(4 × total number of plants), PV = (DSI in control – DSI in treatment) × 100/DSI in control. For each treatment in the A1 and P1 experiments, the marketable yield of Chinese cabbage was recorded as grams of fresh weight per plant. The data for the IR, DSI, PV, and shoot fresh weight were compared according to Duncan’s multiple range test, and a pairwise comparison test was performed. A *p* < 0.05 denoted statistical significance in all tests.

### The Population of *Pcc* E1 Pathogen on the Leaves of Chinese Cabbage in the Greenhouse Assays

The transplanted plants were selected and collected to determine the population of the *Pcc* pathogen on the leaves of Chinese cabbage. On harvest day (ca. 70 days) in the A2 and P2 experiments, three asymptomatic leaves of Chinese cabbage were collected from each plant with a symptomatic score ≤3. Petioles of asymptomatic leaves from each transplanted plant were collected and pooled for the pathogenic bacteria enrichment process as follows (Des Essarts et al., 2016): the petioles were washed with sterile water thrice, 10 ml of phosphate buffer (2.7 g/l of Na_2_HPO_4_ ⋅ 12H_2_O, 0.4 g/l of NaH_2_PO_4_ ⋅ 2H_2_O, pH 7.2) was added, the petioles were ground to a homogenate suspension in a shaking incubator (200 rpm, 2 h) at 25°C. Subsequently, 200 μl of homogenate suspension was transferred into 1,800 μl PEB (0.32 g/l of MgSO_4_, 1.08 g/l of (NH_4_)_2_SO_4_, 1.08 g/l of K_2_HPO_4_, and 1.7 g/l of sodium polypectate) liquid medium (Perombelon and Van Der Wolf, 2002; Czajkowski et al., 2010) and incubation was continued under the same conditions for 48 h. The bacterial culture was centrifuged to collect bacteria for total DNA extraction using the HiPure Bacterial DNA Kit (Magen, China). The presence of the *Pcc* pathogen was detected through polymerase chain reaction (PCR) using the following two *Pcc*-specific primers: EXPCCF (5′-GAA CTT CGC ACC GCC GAC CTT CTA-3′) and EXPCCR (5′-GCC GTA ATT GCC TAC CTG CTT AAG-3′) as previously described (Kang et al., 2003; Humphris et al., 2015). Data were analyzed using the Chi-squared test (*p* < 0.05).

### Monitoring the Pathogenic and Biocontrol Population in the Greenhouse Assays

In the course of the A2 and P2 greenhouse assays, the cabbage leaves and rhizosphere soil samples (*n* = 3, each) were collected at the following seven time points: prior to the transplantation of the seedlings (ca. 0 day), inoculation of pathogen *Pcc* E1 alone (ca. 4 days, only soil samples were collected), 4 days after introduction of the biocontrol agent (ca. 8, 15, 22, and 29 days), and harvest day (ca. 70 days). All samples were used to monitor the population of *B. amyloliquefaciens* KC-1. In contrast, only four soil samples (ca. 0, 4, 29, and 70 days) were used to monitor the population of the *Pcc* E1 pathogen. Subsequently, the plant samples were cut and frozen using liquid nitrogen, and were ground to a powder for DNA extraction using the HiPure Bacterial DNA Kit (Magen, China). Soil samples were air-dried for DNA extraction using the HiPure Soil DNA Mini Kit (Magen, China). The concentration and the quality of the extracted DNA were determined using an Ultrospec 2100 pro UV/visible spectrophotometer (Amersham Biosciences, Pittsburgh, PA, United States) and through electrophoresis on agarose gels (1%, w/vol). Each sample was evaluated in triplicate.

Strain-specific primers were designed to monitor the *B. amyloliquefaciens* KC-1 and pathogen *Pcc* strain E1 from the extracted bacterial and soil DNA using quantitative PCR (qPCR). These primers were designed using the software of the CLC Genomics Workbench 5.5 (CLC Bio, Denmark) and Primer premier 5.0 (Premier, Canada). Their specificity was tested using PCR on bacterial strains (Des Essarts et al., 2016). The primers of *B. amyloliquefaciens* KC-1-*gfp* and *Pcc* E1 were designed to amplify a DNA fragment of ca. 137 and 119 bp, respectively. Finally, the primers GFP-F: (5′-CGA CTT TCG GTT ATG GTG TTC A-3′) and GFP-R: (5′-CGT GTA GTT CCC GTC ATC TTT G-3′) were selected for the detection of *B. amyloliquefaciens* KC-1, while the primers E1-F: (5′-AGG TGC AAG CGT TAA TCG GA-3′) and E1-R: (5′-GCC TCT AGC CTG TCA GTT TTG A-3′) were used for the detection of *Pcc* E1.

The qPCR sample mix was prepared as follows. Initially, 12.5 μl of master mix (Takara, Dalian, China) were vortexed with 0.5 μl of the specific forward and reverse primers (10 μM), and 0.5 μl of the ROX reference Dye (Takara, Dalian, China). Extracted DNA (5 μl) from the soil (HiPure Soil DNA Mini Kit, Magen, China) or plant (HiPure Bacterial DNA Kit, Magen, China) samples and 6.0 μl of SDW were added to the qPCR mix. The qPCR was performed as follows: denaturation step (2 min at 95°C), followed by 40 cycles of 15 s at 95°C, 15 s at 59°C, and 30 s at 72°C. The fluorescence was measured after each cycle. A melting curve analysis was performed at the end of the PCR run (15 s at 95°C, 60 s at 60°C, and 15 s at 95°C) to ensure the amplification of only one PCR product. Data analysis was performed using the StepOne software v2.3 (ABI, PE Applied Biosystems, United States). The relative number of DNA copies per grams/sample was calculated as previously described (Des Essarts et al., 2016).

## Results

### Characterization and Identification of *B. amyloliquefaciens* KC-1

**FIGURE 1:**
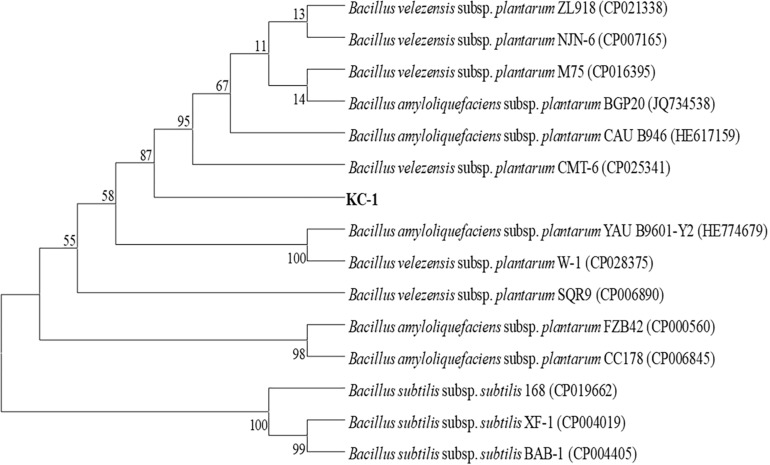
The phylogenetic tree based on partial *gyr*B nucleotide sequences using the neighbor-joining method and showing the position of the KC-1 strain among the ecotypes of *B. amyloliquefaciens*. Bootstrap values of 1,000 replications are shown at the branch points. The accession numbers of the sequences obtained from the National Center for Biotechnology Information database are indicated in parentheses.

KC-1 was found to be a typical *Bacillus* (Logan and De Vos, 2009). When grown on LB agar medium, it formed mucous wrinkles. Under the microscope, the KC-1 cells were rod-shaped and formed ellipsoidal endospores. Furthermore, physiological and biochemical analyses revealed that the KC-1 strain was motile, Gram-positive, and oxidase- and catalase-positive. Meanwhile, the *gyr*B gene sequence of KC-1 (1173 bp) showed 99% identity with *B. amyloliquefaciens* (Figure 1). The sequence was deposited in the GenBank database NCBI (Accession No. MH973156).

### *In vitro* Antibacterial Activity

**FIGURE 2:**
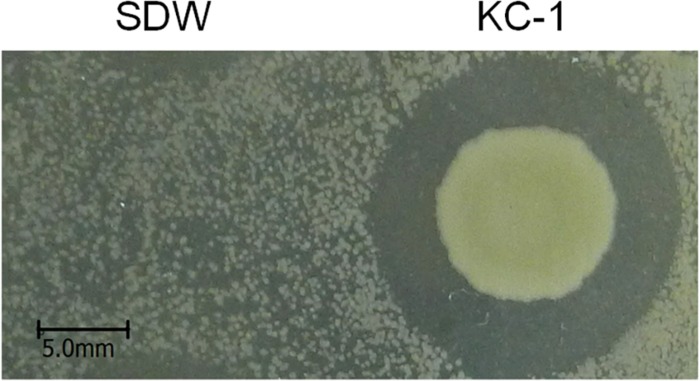
*Bacillus amyloliquefaciens* KC-1 inhibiting the growth of *Pcc* E1.

Clear growth inhibition zones were observed, indicating that *B. amyloliquefaciens* KC-1 exerted a strong effect against *Pcc* E1 on agar plates (Figure 2) previously inoculated with 200 μl *Pcc* E1 cell suspension (10^6^ CFU/ml).

### Evaluation of the Biocontrol Potential of *B. amyloliquefaciens* KC-1

**FIGURE 3:**
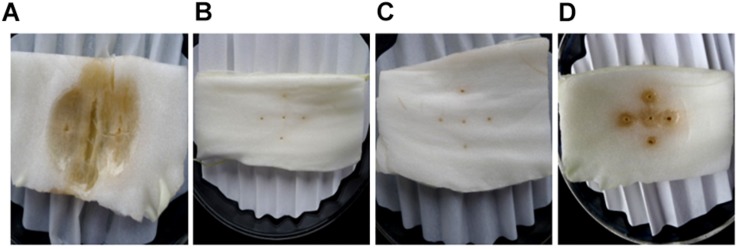
Comparison of the ability of *B. amyloliquefaciens* KC-1 to macerate the petiole tissue of Chinese cabbage. The petiole slices were inoculated with *Pcc* E1 **(A)**, SDW **(B)**, KC-1 **(C)**, or *Pcc* E1++ KC-1 **(D)** suspension (from left to right in the panel).

**FIGURE 4:**
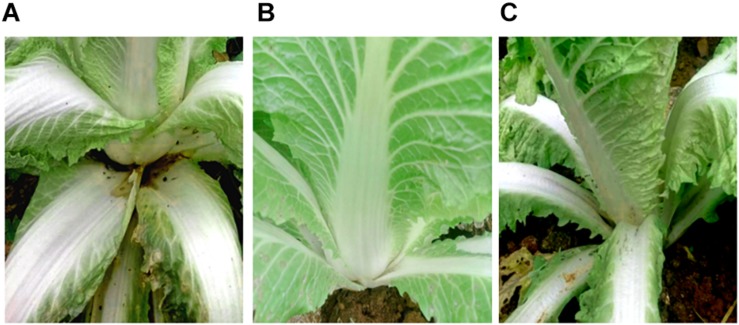
Biocontrol activity assay for soft rot caused by pathogen *Pcc* E1. **(A)** Chinese cabbage inoculated with *Pcc* E1 alone. **(B)** Chinese cabbage inoculated with inactivated *B. amyloliquefaciens* KC-1 alone. **(C)** Chinese cabbage co-inoculated with a mixed inoculation solution (*B. amyloliquefaciens* KC-1 and *Pcc* E1, 1:1 ratio).

SDW and *Bacillus amyloliquefaciens* KC-1 did not negatively affect the Chinese cabbage, following inoculation onto the sterilized surface of healthy tissues. There were no change observed on the tested tissues of petioles (Figures 3B,C). The pathogen *Pcc* E1 caused symptoms of soft rot (strong tissue maceration) on the cabbage (Figure 3A and Table 2). In comparison with pure *Pcc* E1 alone, only slight maceration was observed (Figure 3D and Table 2) following the co-inoculation of petiole slices with a mixed inoculant solution (i.e., *Bacillus* and pathogen at a 1:1 ratio). Furthermore, 3 days after inoculation with the aforementioned mixed inoculation solution, there were no symptoms or only slight symptoms of soft rot observed on the petioles of Chinese cabbage in the greenhouse, compared with control (Figure 4).

**TABLE 2:** Biocontrol assay for soft rot caused to petiole tissue of Chinese cabbage by *Pcc* E1.

**Treatment**	**Maceration area (cm^2^)**	**Percentage of maceration^A^ (%)**
SDW	0^c^	0^c^
KC-1	0^c^	0^c^
*Pcc* E1	10.99 ± 1.08^a^	44.37 ± 2.04^a^
*Pcc* E1+++KC-1	1.85 ± 0.5^b^	7.41 ± 2.14^b^

*^*A*^Percentage maceration = the weight of macerated tissue after inoculation × 100/the weight of tissue before inoculation. Different letters above the bars indicate that the differences are significant (*P* < 0.05) using Duncan’s multiple range test. Values represent the mean of four replications by checking 12 tissues.*

### *In vitro* Influence of *B. amyloliquefaciens* KC-1 on the Growth of *Pcc* E1

**FIGURE 5:**
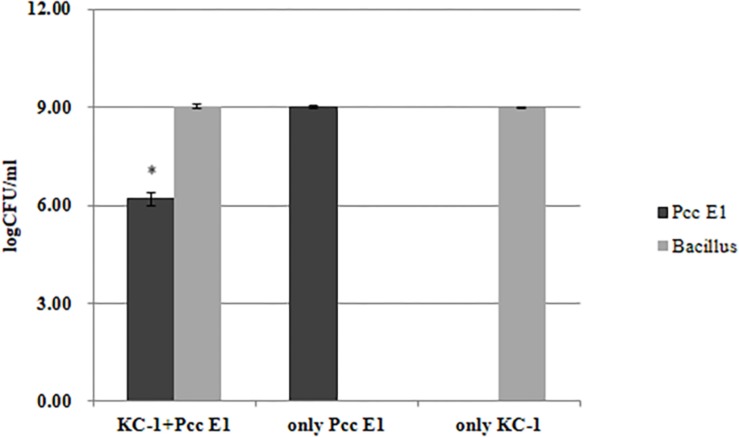
Growth of *Pcc* E1 and *B. amyloliquefaciens* KC-1 in co-culture assays after 12 h. The data were analyzed using the independent *t*-test. Values represent the mean of three replications. Bars indicate the standard deviation of the mean. The asterisk indicates values that were significantly different (*p* < 0.05).

The distinct colony morphologies of *B. amyloliquefaciens* KC-1 (i.e., opaque and large) and *Pcc* E1 (i.e., translucent and small) were used to quantify their growth in the co-culture experiments. *Pcc* E1 showed ∼10^9^ CFU/ml when cultured alone and ∼10^7^ CFU/ml in co-culture, indicating that its growth was inhibited by *B. amyloliquefaciens* KC-1 (Figure 5). Meanwhile, *B. amyloliquefaciens* KC-1 showed ∼10^9^ CFU/ml under both culture conditions.

### Characterization of Potential Antibacterial Traits

Several traits potentially involved in antimicrobial activities were detected *in vitro*. Tested antibiosis traits included presence of antibiotic genes (Table 3). Grounded on the results of PCR analysis, strain KC-1 showed the presence of genes involved in the biosynthesis of the polyketides difficidin (*dfnA*), bacillaene (*baeA*), and macrolactin (*mlnA*) and the dipeptide bacilysin (*bacA*). The results indicated that strain KC-1 possess antibacterial activities.

**TABLE 3:** Evaluation of antibacterial traits of *B. amyloliquefaciens KC-1 in vitro*.

**Polyketide genes**			**Dipeptide gene**
**Difficidin**	**Bacillaene**	**Macrolactin**	**Bacilysin**
+	+	+	+

### GFP Tagging of the *B. amyloliquefaciens* KC-1

**FIGURE 6:**
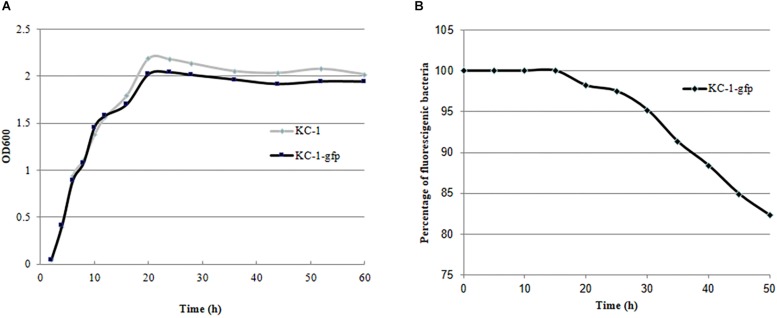
The stability and growth characteristics of the *B. amyloliquefaciens* KC-1 GFP-tagged strain. **(A)** Growth curve of the *B. amyloliquefaciens* KC-1 GFP-tagged strain and its wild type. **(B)** Assessment of stability of the *B. amyloliquefaciens* KC-1 GFP-tagged strain.

Validation of the KC-1-*gfp* transformants was performed by measuring their GFP fluorescence using photoelectric refractometer (data not shown). The growth characteristics in LB broth (Figure 6A) and its antagonistic ability against the *Pcc* E1 strain on LB agar plates (data not shown) did not exhibit a significant difference between the KC-1-*gfp* and KC-1 wild type, suggesting that the normal metabolism of KC-1 was not disrupted by the presence of the plasmid. The stability of the pHT01-P43GFPmut3a plasmid in KC-1-*gfp* was evaluated through continuous culture in LB broth without antibiotic treatment. After a 60-h incubation on LB agar plates only ∼18% of the KC-1-*gfp* culture lost the plasmid (Figure 6B), indicating that the KC-1-*gfp* strain could be used in the subsequent prolonged colonization experiments.

### Promotion of Seedling Growth by the Bio-Organic Substrate

**FIGURE 7:**
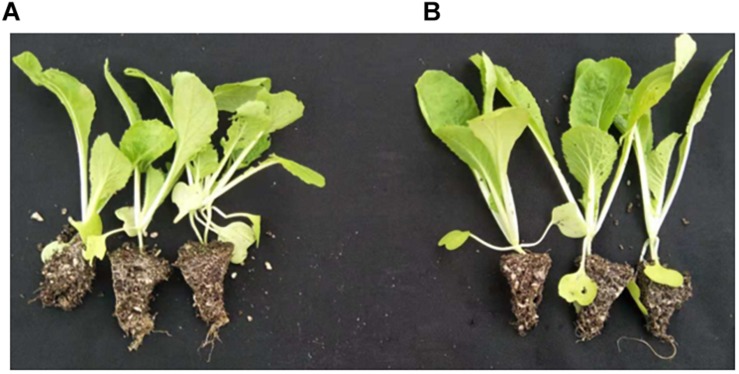
Promotion of growth using the bio-organic substrate. **(A,B)** Chinese cabbage seedlings cultivated with common organic substrate and bio-organic substrate containing *B. amyloliquefaciens* KC-1, respectively.

The shoot fresh and dry weight, as well as the height, of the Chinese cabbage seedlings grown in a bio-organic substrate for 25 days was significantly increased by 52.8, 32.4, and 17.45%, respectively, compared with the control (Figure 7 and Table 4). In contrast, the values of the root fresh and dry weight did not exhibit a significant difference between the two treatments.

**TABLE 4:** The efficacy of different treatments on promoting seedling growth in floating-seedling system.

**Treatment^a^**	**Shoot fresh weight (g/10 plants)**	**Root fresh weight (g/10 plants)**	**Shoot dry weight (g/10 plants)**	**Root dry weight (g/10 plants)**	**Shoot height (cm)**
OS	4.3	1.2	0.4	0.1	7.9
BIO	6.6^*^	1.4	0.6^*^	0.1	9.3^*^

*^*a*^OS, organic substrate; BIO, bio-organic substrate containing *B. amyloliquefaciens* KC-1. The data were analyzed using the independent *t-*test. Values represent the mean of three replications by checking 30 plants. Asterisk indicates values that are significantly different (*p* < 0.05).*

### Suppression of the Severity of Soft Rot by *B*. *amyloliquefaciens* KC-1 in the Greenhouse Assays

Four independent experiments (i.e., A1, A2, P1, and P2) were performed in two greenhouses over a period of 2 years (Table 5). Chinese cabbage seedlings were grown in greenhouse soil or bio-organic substrate using the floating-seedling system for 25 days. Subsequently, they were transplanted and used in the greenhouse under different conditions. In all these experiments, there were no symptoms observed under the water control condition in plants treated without pathogen (data not shown). Similarly, there was no significant difference in soft rot symptoms between the plants grown using the floating-seedling system in the absence of *B. amyloliquefaciens* KC-1 and those directly sown with *Pcc* alone (Table 5). Following treatment with *B. amyloliquefaciens* KC-1, there was a tendency toward reduction in the severity of disease compared with the control. In addition, plants grown in the presence of the biocontrol agent both in the growth stage of the floating-seedling system and the greenhouse significantly reduced the severity of disease compared with application in one of the two stages (Table 5).

**TABLE 5:** Disease suppression of different treatments against bacterial soft rot of Chinese cabbage in greenhouse assays.

		**Anning**				**Panlong**			
**Year**	**Treatment^A^**	**Incidence rate^B^ (%)**	**Disease severity index^C^**	**Protection value^D^**	**Marketable yield [kg (m^2^)^–1^]**	**Incidence rate (%)**	**Disease severity index**	**Protection value**	**Marketable yield [kg(m^2^)^–1^]**
2015	CK	100.0 ± 0.0^a^	70.1 ± 2.4^a^		10.3 ± 2.5^c^	94.4 ± 4.8^a^	67.4 ± 3.2^a^		9.1 ± 2.6^bc^
	OS + Water	100.0 ± 0.0^a^	67.4 ± 1.2^a^	3.9 ± 0.21^d^	10.0 ± 1.8^c^	97.2 ± 4.8^a^	72.2 ± 3.2^a^	0	5.9 ± 0.9^c^
	OS + KC-1	97.2 ± 4.8^a^	35.4 ± 2.1^c^	49.5 ± 3.1^b^	19.6 ± 0.6^a^	77.8 ± 9.6^b^	27.8 ± 4.3^c^	58.8 ± 6.0^a^	23.3 ± 2.4^a^
	BIO + Water	100.0 ± 0.0^a^	49.3 ± 3.2^b^	29.7 ± 4.7^c^	14.7 ± 4.0^b^	100.0 ± 0.0^a^	54.9 ± 4.3^b^	18.5 ± 6.0^b^	10.9 ± 1.7^b^
	BIO + KC-1	66.7 ± 14.4^b^	22.2 ± 3.2^d^	68.3 ± 4.7^a^	22.7 ± 1.0^a^	72.2 ± 12.7^b^	27.1 ± 3.6^c^	59.8 ± 5.0^a^	23.2 ± 2.1^a^
2016	CK	100.0 ± 0.0^a^	71.5 ± 6.4^a^		ND^E^	100.0 ± 0.0^a^	65.9 ± 4.8^a^		ND
	OS + Water	97.2 ± 4.8^a^	63.2 ± 5.2^a^	11.6 ± 1.9^d^	ND	100.0 ± 0.0^a^	66.0 ± 5.2^a^	0	ND
	OS + KC-1	80.6 ± 4.8^b^	27.8 ± 4.3^c^	61.1 ± 6.9^b^	ND	80.6 ± 4.8^b^	26.4 ± 3.2^c^	59.9 ± 4.8^a^	ND
	BIO + Water	88.9 ± 4.8^ab^	43.1 ± 5.2^b^	39.7 ± 8.3^c^	ND	94.4 ± 4.8^a^	53.5 ± 4.3^b^	18.8 ± 6.6^b^	ND
	BIO + KC-1	50.0 ± 14.4^c^	16.7 ± 4.2^d^	76.6 ± 6.6^a^	ND	44.4 ± 4.8^c^	21.5 ± 5.2^c^	67.3 ± 7.9^a^	ND

*^*A*^CK, the treatment where the plants grown in infected soil by directly sowing; OS + Water, seedlings grown in organic substrate and infected with *Pcc* E1 alone after translating; OS + KC-1, seedlings grown in organic substrate and infected with *Pcc* E1 and *B. amyloliquefaciens* KC-1 after translating; BIO + Water, seedlings grown in bio-organic substrate and infected with *Pcc* E1 alone after translating; BIO+KC-1, seedlings grown in bio-organic substrate and infected with *Pcc* E1 and *B. amyloliquefaciens* KC-1 after translating. ^*B*^Incidence rate = Σ(the number of diseased plant) × 100/total plant number). ^*C*^Disease severity index = Σ(scale × diseased plant number) × 100/(4 × total plant number). ^*D*^Protection value = (DSI in control – DSI in treatment) × 100/DSI in control. Different letters above the bars indicate that the differences are significant (*P* < 0.05) using Duncan’s multiple range test. ^*E*^ND, not determined.*

### Limitation of Spread of the Pathogen *Pcc* on the Leaves of Chinese Cabbage

**FIGURE 8:**
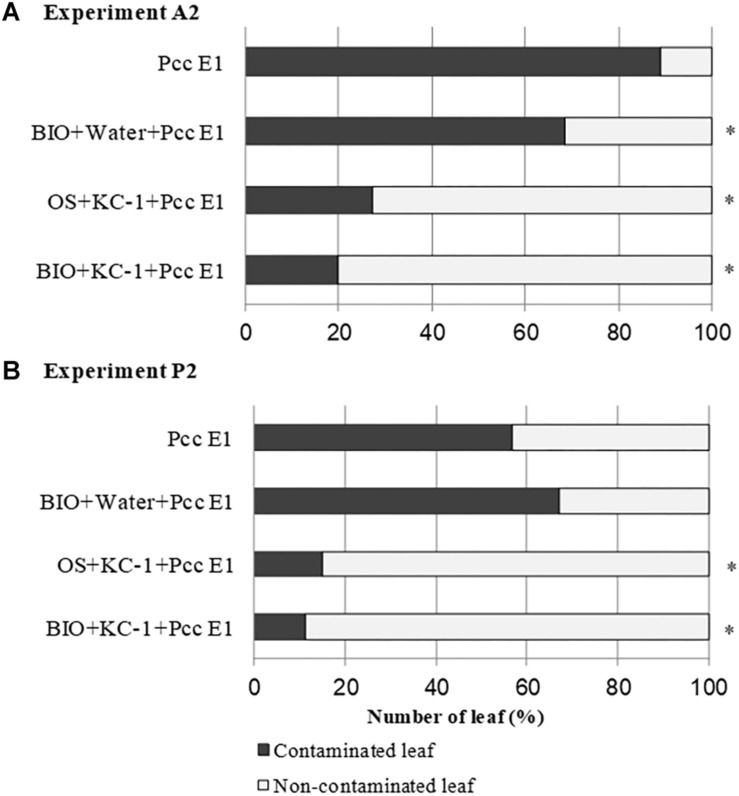
Propagation of pathogen *Pcc* E1 in the petioles of Chinese cabbage. Detection of *Pcc* E1 through PCR was conducted in asymptomatic petioles obtained from the A2 **(A)** and P2 **(B)** experiments. Plants were cultivated in the presence (+) or absence (–) of *B. amyloliquefaciens* KC-1 in two growth stages (floating-seedling system and greenhouse). OS, organic substrate; BIO, bio-organic substrate containing *B. amyloliquefaciens* KC-1. Significant reductions (Chi-squared test; *p* < 0.05) in the presence of *Pcc* E1 are indicated by asterisks.

On harvest day (ca. 70 days) of the A2 and P2 experiments, asymptomatic outer leaves were collected from the plant with a symptomatic score ≤3 and screened for *Pcc* through PCR (Figure 8). In experiment A2, all the combinations treated with *B*. *amyloliquefaciens* KC-1 exhibited reduced dissemination of the pathogen in the asymptomatic Chinese cabbage leaves. In experiment P2, a significant decrease in the pathogen was recorded following the application of *B*. *amyloliquefaciens* KC-1 in the growth stage in the greenhouse only, and combination of both floating-seedling system and greenhouse.

### Limitation Survival and Multiplication of the Pathogen *Pcc* in the Rhizosphere Soil of Chinese Cabbage

**FIGURE 9:**
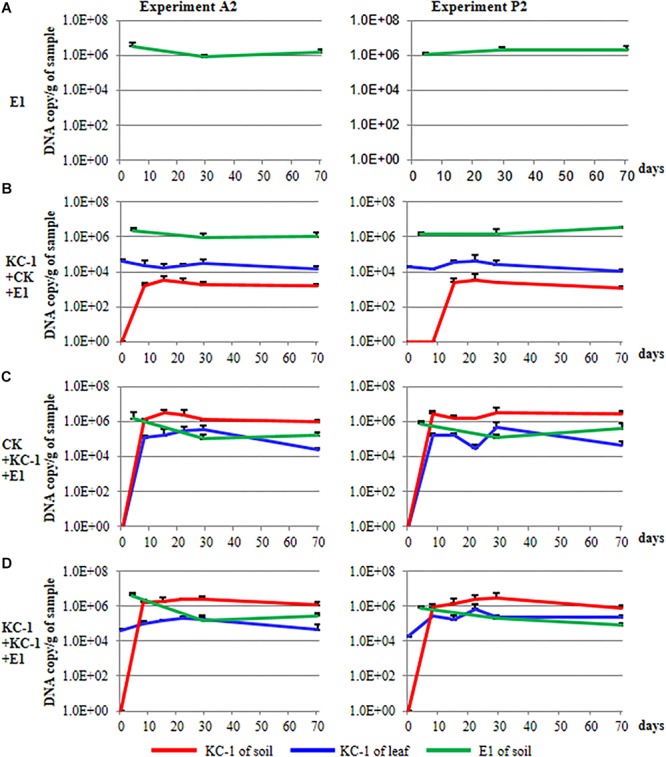
Population dynamics of the biocontrol and pathogenic strains in Chinese cabbage. **(A)** The plants were cultivated using the floating-seedling system with OS and inoculated with *Pcc* E1 alone after transplantation. **(B)** Plants were cultivated using the floating-seedling system with BIO and inoculated with *Pcc* E1 alone after transplantation. **(C)** Plants were cultivated using the floating-seedling system with OS and inoculated with *Pcc* E1 and KC-1 after transplantation. **(D)** Plants were cultivated using the floating-seedling system with BIO and inoculated with *Pcc* E1 and KC-1 after transplantation.

During the course of the A2 and P2 experiments, the population of *Pcc* E1 in the rhizosphere of Chinese cabbage was measured through qPCR using specific primers (Figure 9). The population size of *Pcc* E1 was attained at a level of 10^4^–10^6^ DNA copies/g of dry soil in all conditions after introduction of the pathogen (green lines in Figure 9). The *B*. *amyloliquefaciens* KC-1 applied in the greenhouse reduced one or two orders of magnitude of *Pcc* E1 compared with the control.

### Population Dynamics of *B*. *amyloliquefaciens* KC-1 in the Greenhouse Assays

During the course of the A2 and P2 experiments, the population dynamics of *B*. *amyloliquefaciens* KC-1 in the cabbage leaves and rhizosphere was detected, exhibiting similar patterns (Figure 9). Notably, there was no DNA copy detected in the soil samples collected for all treatments prior to transplantation (i.e., 0 day) in the greenhouse. This finding confirmed the absence of *B*. *amyloliquefaciens* KC-1 in the soil prior to the application of the strain (red lines in Figure 9). Cabbage grown in the presence of KC-1 by floating-seedling system and without KC-1 in greenhouse (bio-organic substrate plus water), we detected a consistent cell concentration approximately 10^4^ DNA copies/g of petiole. In the other treatments of bio-organic substrate plus *B*. *amyloliquefaciens* KC-1 and organic substrate plus *B*. *amyloliquefaciens* KC-1 was decreased by two orders of magnitude after its application (blue lines in Figure 9). Meanwhile, while biocontrol agent was introduced by spraying in the greenhouse, the population size of KC-1-*gfp* was attained at a level of 10^6^ DNA copies/g of dry soil, three orders of magnitude higher than the treatment with biocontrol agent only inoculated in the floating-seedling system (bio-organic substrate plus water, red lines in Figure 9).

## Discussion

Studies have reported the use of *Bacillus* sp. as biocontrol strains against pathogens associated with bacterial soft rot (Yang et al., 2011; Czajkowski et al., 2012; Des Essarts et al., 2016; Garge and Nerurkar, 2017; Gerayeli et al., 2018). However, certain *Bacillus* sp. have also been shown to cause bacterial soft rot in various vegetables, including the onion (Hwang et al., 2012), arrowhead (Zhong et al., 2015), and potato (Wang et al., 2017). Moreover, a study reported that *Bacillus* sp. cause severe decay in the potato, and may effectively control bacterial soft rot in potatoes, green peppers, and Chinese cabbages (Zhao et al., 2013). In the present study, *B*. *amyloliquefaciens* KC-1 was successfully isolated and identified. We revealed that this strain did not induce symptoms of soft rot on the tested plant tissues. However, we also demonstrated their *in vitro* and *in vivo* activity against the *Pcc* E1 strain. This result was also validated using PCR analysis of KC-1 showing the presence of genes involved in the biosynthesis of the polyketides difficidin, bacillaene, and macrolactin and the dipeptide bacilysin.

Real-time PCR is able to detect and quantify microorganisms in plant tissues and soil samples (Des Essarts et al., 2016). Considering the possible high abundance of native bacteria belonging to *B*. *amyloliquefaciens* in the rhizosphere soil of plants, the *B. amyloliquefaciens* KC-1-*gfp* was used in this study instead of its wild type. The qPCR results confirmed that *B*. *amyloliquefaciens* KC-1 can colonize the rhizosphere soil and leaves of Chinese cabbage. High densities of *B*. *amyloliquefaciens* KC-1-*gfp* were recovered in the rhizosphere soil prior to harvest. Colonization of the rhizosphere by the biocontrol agent is critical for the effective control of phytopathogens (Compant et al., 2005; Cao et al., 2011; Diallo et al., 2011). This suggests that the presence of high densities of *B*. *amyloliquefaciens* KC-1 strain in the rhizosphere soil is a prerequisite for suppressing the infection caused by the soil-borne pathogen *Pcc*. Biocontrol agents control soil-borne diseases through interference with their biological antibiotic responses, competition for niches and nutrients in the root and location of the lesion, and induction of systemic resistance in host plants (Bais et al., 2004; Compant et al., 2005; Haas and Défago, 2005; Cao et al., 2011). Recently, a study showed that the population of the pathogen and symptoms in the rhizosphere soil of *Ralstonia solanacearum* were significantly decreased following the introduction of a *Bacillus* sp. (Tan et al., 2013). Of note, the introduction of *Pseudomonas* sp. reduced the blackleg and symptoms of soft rot symptoms; however, it did not succeed in limiting the concentration of the pathogen in the rhizosphere soil (Des Essarts et al., 2016). In comparison, the results of the present study clearly demonstrated that *B. amyloliquefaciens* KC-1 competes with the pathogen in the rhizosphere by significantly decreasing the pathogen copies in the rhizosphere soil and leaves of inoculated Chinese cabbage. This may be attributed to the adaptability of the *Bacillus* sp. in the rhizosphere and host plant. The biocontrol strains examined in this study may play the role of a “defender” against infections in plant by directly limiting the survival and transmission of the pathogen in the plant rhizosphere and tissues.

The biocontrol agents associated with the roots may also prevent *Pcc* from invading the roots through antibiosis (Dong et al., 2004; Des Essarts et al., 2016; Tsuda et al., 2016; Garge and Nerurkar, 2017). In this study, *B. amyloliquefaciens* KC-1 induced a significant suppression of growth and reduction in the number of colonies of *Pcc* in plate assay and co-culture assay, respectively. These findings indicate the production of diffusible antibacterial compounds. Meanwhile, the percentage of *Pcc*-carrying asymptomatic leaves in Chinese cabbage inoculated with *B. amyloliquefaciens* KC-1 was reduced both directly and indirectly, suggesting that the transmission of *Pcc* to inner leaves was limited. The *Bacillus* sp. may act against pectinolytic bacteria by competing for nutrients and by producing signal molecules to disrupt the pectinolytic bacteria (Molina et al., 2003; Dong et al., 2004; Uroz et al., 2009; Garge and Nerurkar, 2017), and various antimicrobial molecules (Diallo et al., 2011) (e.g., siderophores, volatile compounds, antibiotics, and hydrogen peroxide).

The floating-seedling system – widely used in agriculture – assists in the growth of plant seedlings under non-pathogenic conditions (Yan et al., 2003; Sung and Hong, 2010). The organic substrate provides nutrients to ensure the survival and persistence of growth in the floating-seedling system. Recently, we reported that clubroot – one of the most destructive diseases of Chinese cabbage – may be controlled through seedling growth using the floating-seedling system in the absence of a biocontrol agent (He et al., 2019). However, there was no significant reduction observed in the symptoms of soft rot following the application of this method compared with direct seeding. In this study, we introduced a new biocontrol product – termed bio-organic substrate – by mixing the traditional organic substrate with *B*. *amyloliquefaciens* KC-1. Use of the bio-organic substrate in the floating-seedling system promoted growth of seedlings compared with the conventional organic substrate. In the greenhouse, three times introduction of *B*. *amyloliquefaciens* KC-1 culture to the soil reduced the incidence and severity index of soft rot in Chinese cabbage. The bio-organic substrate in the floating-seedling system alone reduced the density of *B*. *amyloliquefaciens* KC-1-*gfp* (10^4^–10^5^ copy/g) in plants. The disease incidence was not reduced but could decrease the DSI, which may be due to the plant niches occupied by the strain KC-1 before *Pcc* E1 when the seedlings were grown in bio-organic substrate. Chinese cabbage seedlings grown in bio-organic substrate for 25 days, transplanted to the greenhouse, and treated with bacterial culture, exhibited a reduced incidence and severity index of soft rot, as well as impaired transmission of the *Pcc* strain in the cabbage leaves. Thus, colonization of Chinese cabbage by biocontrol bacteria prior to the introduction of *Pcc* may protect the plant from soft rot and suppress pathogen survival in the rhizoplane soil. Moreover, these results showed that *B. amyloliquefaciens* KC-1 – applied in an appropriate manner – may effectively manage soft rot in Chinese cabbage. However, field experiments are warranted to verify the competition and persistence of *B. amyloliquefaciens* KC-1 under natural conditions and its contribution to the reduction of disease symptoms and transmission of *Pcc*.

## Conclusion

In conclusion, *B. amyloliquefaciens* KC-1 effectively reduced the symptoms of bacterial soft rot in Chinese cabbage and transmission of the *Pcc* pathogen under greenhouse conditions. Suitable carrier materials and methods are necessary for the function of this antagonist in the soil and its application in the field, providing a theoretical basis for the development of a bio-organic substrate. Use of a new bio-organic substrate at the initiation of the floating-seedling system and introduction of the *B. amyloliquefaciens* KC-1 strain to plants three times after transplantation resulted in good control of soft rot. This was achieved through successful colonization of the plant rhizosphere and tissues, consequently leading to a marked decrease in the population of the pathogen in the rhizosphere and transmission to the leaves. Furthermore, research investigating the bio-antibacterial mechanisms of *B. amyloliquefaciens* KC-1 (i.e., quorum quenching, competition for nutrients, and induction of resistance in host plants) is currently ongoing in our laboratory.

## Data Availability

The raw data supporting the conclusions of this manuscript will be made available by the authors, without undue reservation, to any qualified researcher.

## Author Contributions

WC, PJH, PFH, YH, and YW designed the experiments and revised the manuscript. WC, PJH, PBH, PFH, XL, LY, BW, and SM performed the experiments and analyzed the data. WC, PJH, PFH, SM, and YH wrote the draft. All authors viewed the draft of manuscript.

## Conflict of Interest Statement

The authors declare that the research was conducted in the absence of any commercial or financial relationships that could be construed as a potential conflict of interest.

## Footnotes

1 http://blast.ncbi.nlm.nih.gov/Blast.cgi
